# *Auricularia auricular* Adsorbs Aflatoxin B1 and Ameliorates Aflatoxin B1-Induced Liver Damage in Sprague Dawley Rats

**DOI:** 10.3390/foods12142644

**Published:** 2023-07-08

**Authors:** Dan Xu, Minmin Huang, Jiao Lei, Hongxin Song, Liangbin Hu, Haizhen Mo

**Affiliations:** School of Food Science and Engineering, Shaanxi University of Science and Technology, Xi’an 710021, China; xudan@sust.edu.cn (D.X.); hmm@sust.edu.cn (M.H.); leijiao1120@126.com (J.L.); songhx@sust.edu.cn (H.S.); hulb@sust.edu.cn (L.H.)

**Keywords:** AFB1, *Auricularia auricular*, adsorption, liver protection

## Abstract

Aflatoxin B1 (AFB1), as a class I carcinogen, poses a substantial health risk to individuals. Contamination of food sources, particularly grains and nuts, with *Aspergillus flavus* (*A. flavus*) contributes to the prevalence of AFB1. The impact of global warming has spurred research into the development of AFB1 prevention technologies. While edible fungi have shown potential in detoxifying AFB1, there is a scarcity of literature on the application of *Auricularia auricular* (*A. auricular*) in this context. This study aimed to investigate the ability and underlying mechanism of *A. auricular* mycelia to adsorb aflatoxin B1, as well as evaluate its protective effects on the AFB1-induced liver damage in SD rats. Additionally, the effects of temperature, time, pH, and reaction ratio on the adsorption rate were examined. Combining thermodynamic and kinetic data, the adsorption process was characterized as a complex mechanism primarily driven by chemical adsorption. In SD rats, the *A. auricular* mycelia exhibited alleviation of AFB1-induced liver damage. The protective effects on the liver attributed to *A. auricular* mycelia may involve a reduction in AFB1 adsorption in the intestine, mitigation of oxidative stress, and augmentation of second-phase detoxification enzyme activity. The adsorption method for AFB1 not only ensures safety and non-toxicity, but also represents a dietary regulation strategy for achieving effective defense against AFB1.

## 1. Introduction

Aflatoxin B1 (AFB1), which is the most highly toxic mycotoxin, is predominantly produced by *Aspergillus flavus* and *Aspergillus parasiticus* [1,2], and can frequently be found in foods such as milk, rice, corn, peanuts, oils, and soybeans [3,4]. It can cause damage to our liver, lungs, heart, and kidneys, and it shows immunosuppressive properties, such as inhibiting cell-mediated immune reactions, decreasing natural killer cytolysis, and suppressing macrophage function [5,6,7,8]. Currently, it has been listed as a class I carcinogen by the World Health Organization (WHO) [9,10,11,12]. Its carcinogenic action requires exertion by metabolic activation [13]. Once adsorbed, aflatoxin undergoes metabolism by hepatic cytochrome P450 enzymes, resulting in the formation of AFB1-8,9-epoxide (AFBO) [14]. AFBO is considered pivotal in the toxicity of aflatoxins, and it undergoes an adduct reaction with DNA, causing DNA damage and genetic mutations [15].

To reduce the exposure risk of aflatoxin in the diet, numerous approaches have been investigated for the removal of AFB1. These approaches can be broadly categorized into physical, chemical, and biological control methods [16,17]. Considering safety and efficiency, biological detoxification is emerging as a favorable approach for the removal of aflatoxins [17,18]. The application of microorganisms used in the food industry to remove aflatoxins, including lactic acid bacteria, yeast, and edible fungi, has been reported in a lot of research [19,20,21,22]. It is found that AFB1 can be effectively degraded by enzymes extracted from edible fungi, such as *Armillariella tabescens*, *Pleurotus ostreatus*, *Plerotus eryngii*, and *Agaricus blazei* [23,24,25].

In addition, many studies have shown that AFB1 can not only be degraded by microorganisms through enzymatic degradation, but also adsorbed by the cell wall components of microorganisms. To better understand the role of fungal components in AFB1 removal activity, the ability of *Bjerkandera adusta* (*B. adusta*) to remove AFB1 was analyzed after enzymatic, physical, and chemical treatments to degrade or change the fungal cellular components. The results suggest that AFB1 was removed by its binding onto the cell wall components of *B. adusta* [26].

In the preliminary research, we studied the ability of seven edible fungi (*Lentinus edodes*, *Flammulina velutiper*, *Pleurotus ostreatus*, *Hericium erinaceum*, *Armillariella mellea*, *Auricularia auricular*, and *Auricularia polytricha*) to remove AFB1, and found that *A. auricular* has the strongest ability to remove AFB1, with a removal rate of 88.2%. Interestingly, the efficacy of *A. auricular* for AFB1 removal is primarily attributed to its adsorption capabilities, rather than biodegradation. Therefore, edible fungi have demonstrated potential as AFB1 adsorbents, due to their edibility and high adsorption capacity [19]. Based on the composition and structural characteristics of *A. auricular*, it contains a lot of dietary fiber that is not digested by the body and is able to adsorb heavy metals, tetracycline, and other pollutants [27,28,29].

Furthermore, *A. auricular* contains many pharmacologically active ingredients, which have been emphasized for use in various biological activities, such as hepatoprotective and antioxidative action [30,31]. Therefore, we speculate that *A. auricular* can effectively alleviate the liver damage caused by AFB1. On the one hand, *A. auricular* prevents AFB1 from being adsorbed into the intestine by binding the toxin; on the other hand, the active ingredients contained in *A. auricular* may activate the body’s immune function and protect the liver from oxidative damage caused by AFB1.

To comprehensively assess the detoxification effect of *A. auricular* on AFB1, this research systematically investigated the impact of various parameters, such as temperature, pH, and reaction ratio, on the adsorption of AFB1 by *A. auricular* *in vitro*. Subsequently, the adsorption process was characterized by analyzing the kinetics and thermodynamics of adsorption. Finally, the protective effect of *A. auricular* against AFB1-induced liver injury was evaluated in an *in vivo* model.

## 2. Materials and Methods

### 2.1. Strains, Chemicals, and Reagents

*A. auricular* was purchased from Xixiang Edible Fungi Research Institution (Xixiang, Shaanxi Province) and stored at 4 °C on potato dextrose agar (PDA, BD) medium. AFB1 and dimethyl sulfoxide (DMSO) were purchased from Sigma Company (Ronkonkoma, NY, USA). All kits (Superoxide dismutase (SOD) kit, glutathione S-transferase (GST) kit, etc.) were purchased from Nanjing Jiansheng Biological Engineering Co., Ltd. (Nanjing, China). Other chemicals and reagents were purchased from Sinopharm Chemical Reagent Co., Ltd. (Shanghai, China). And 10 mg/mL AFB1 stock solution was prepared by dissolving 10 mg AFB1 powder in 1 mL of methanol solution.

### 2.2. Assessment of Adsorption Capacity under Different Pretreatments

*A. auricular* was cultured in PDA medium at 28 ± 2 °C until all over the plate. Subsequently, the mycelia were transferred to potato dextrose broth (PDB) medium, and incubated at 28 ± 2 °C under continuous shaking at 120 r/min in the absence of light, for a period of 4–7 days. The mycelia were then separated through vacuum filtration. The mycelia were divided into 4 groups, each containing 2 g, and individually combined with 2 mL of phosphate-buffered saline (PBS) buffer to form separate *A. auricular* suspensions. The four groups were subjected to different pretreatment procedures: (1) heated at 121 °C for 20 min; (2) pre-freezing at −20 °C for 12 h, followed by repeated cycles of freezing after dissolution for 12 h, repeated for 3 times; (3) freeze-drying for 12 h after pre-freezing at −20 °C; or (4) a blank control, where the *A. auricular* suspension was not subjected to any treatment. Samples with different pretreatments (the above 4 groups) were added to up to 0.4 μg/mL of AFB1, then cultured at the same conditions for 12 h. Finally, the free toxins were quantified using high-performance liquid chromatography (HPLC).

The residual AFB1 was detected according to the method by Xu [32]. AFB1 present in the 1 mL filtrate sample was extracted using 3 mL of chloroform, followed by drying using nitrogen gas. The resulting AFB1 extract was then dissolved in 100 µL of trifluoroacetic acid and 200 µL of *n*-hexane, and kept in a dark environment at 40 °C for 15 min. The derivative obtained was subsequently dried using nitrogen gas blowing, and redissolved in 1 mL of acetonitrile–water (30:70; *v*/*v*). The AFB1 derivative solution was then filtered through a 0.22 µm filter, and quantified using an Agilent HPLC 1200 liquid-chromatograph (Agilent Technologies, Palo Alto, CA, USA) equipped with a C18 column (Diamonsil^®^, 250 mm × 4.6 mm, 5 µm) that was conditioned at 30 °C. The mobile phase consisted of 30% acetonitrile and 70% water, with a flow rate of 1.0 mL/min. Finally, the AFB1 signal was monitored using a fluorescent detector at excitation/emission wavelengths of 360/440 nm.

### 2.3. Effects of Reaction Ratio (Mycelia/AFB1), pH, Temperature and Incubation Time on AFB1 Adsorption Capacity

The effects of various factors on adsorption were investigated using vacuum freeze-dried *A. auricular* powder. A suspension of *A. auricular* was prepared by mixing 0.05 g of mycelia powder with 5 mL of PBS buffer. Different initial concentrations of AFB1 (ranging from 0.0125 to 1.0 μg/mL) were added, and the mixture was cultured for 12 h. The supernatant was collected to determine the residual AFB1. In addition, experiments were conducted by adding AFB1 to a concentration of 0.4 μg/mL, and the adsorption process was studied under various pH levels (ranging from 2 to 8), temperatures (ranging from 20 to 60 °C), and co-culturing times (from 2 to 12 h).

### 2.4. Stability of the Adsorption

The mycelia separated from co-cultured systems were subjected to dissolution using various solvents, including sodium hydroxide (NaOH), acetonitrile (C_2_H_3_N), methanol-acetonitrile mixture (CH_3_OH:C_2_H_3_N = 1:1), and 0.01 mol/L hydrochloric acid (HCl), at 30 °C for 6 h, 160 r/min. Then, the amount of AFB1 in the supernatant was defined as the desorption quantity of the binder.

The experiment was divided into two groups. In the first group, 40 μL AFB1 solution (10 mg/L) was added, then 1 mL artificial gastric juice and artificial intestinal juice were added, respectively. Then, 0.01 g of *A. auricular* mycelia powder was added, and the mixture was co-cultured 37 °C for 0.5–4 h at 200 r/min. After centrifugation at 5000 r/min for 10 min, the supernatant was collected for AFB1 content determination. In the second group, the binders obtained after 4 h of culture were mixed with 1 mL of artificial gastric juice and artificial intestinal juice, and the mixture was shaken at 37 °C for 0.5–4 h at 200 r/min. Finally, the supernatant was collected, and the quantity of AFB1 was determined using HPLC.

### 2.5. Adsorption Kinetics

A quantity of mycelia powder (0.05 g) was subjected to co-cultivation with AFB1 at a concentration of 0.4 μg/mL at 35 °C for 2–12 h. The adsorption of AFB1 was subsequently evaluated using the same experimental procedure. Four different kinetic models, namely the intraparticle diffusion model, pseudo-first-order kinetic model, pseudo-second-order kinetic model, and Elovich model, were chosen for fitting analysis in order to determine the most suitable kinetic model for describing the adsorption process.

(1) $$q_{t}=k_{p}t^{0.5}$$

(2) $$\ln(q_{e}-q_{t})=\ln q_{e}-k_{1}t$$

(3) $$\frac{t}{q_{t}}=\frac{1}{k_{2}q_{e}{}^{2}}+\frac{t}{q_{e}}$$

(4) $$q_{t}=A_{E}+B_{E}\ln t$$

### 2.6. Adsorption Isotherm

A total of 0.05 g mycelia powder and PBS buffer were cultured together to form a system of 5 mL. Then, we set a range of initial concentrations of AFB1 (0.0125–0.4 μg/mL) and tested at 25–45 °C for 8 h, and separately followed the above methods to map adsorption isotherm. The quantity of AFB1 adsorbed per unit of adsorbent can be calculated using the Formula (5):

(5) $$Q_{e}=\frac{\left(C_{o}-C_{e}\right)\times V}{m}$$

where Qe is the adsorption capacity of AFB1 (μg/Kg); Co is the concentration of AFB1 (μg/L); Ce is the equilibrium concentration of AFB1 (μg/L); V is the volume of AFB1 (mL); and M is the mass of added mycelia powder (g).

The Freundlich (6) and Langmuir empirical Formulae (7) were used to fit the adsorption results and describe the adsorption process of AFB1 by *A. auricular* mycelia:

(6) $$Q_{e}=K_{f}\times C_{e}{}^{\frac{1}{n}}$$

(7) $$Q_{e}=\frac{Q_{m}\times K_{L}\times C_{e}}{1+K_{L}\times C_{e}}$$

### 2.7. Adsorption Thermodynamics

The relevant thermodynamic parameters are calculated by the following formulae:

(8) $$K_{OC}=\frac{K_{f}}{W_{OC}}$$

(9) $$\Delta G=-RT\ln K_{d}$$

(10) ΔG=ΔH−TΔS

(11) $$K_{d}=\frac{Q_{e}}{Q_{c}}=\frac{C_{s}}{C_{e}}\times \frac{V}{M}$$

(12) $$\ln K_{d}=\frac{\Delta S}{R}-\frac{\Delta H}{R}\times \frac{1}{T}$$

### 2.8. The In Vivo Evaluation of the Intervention Effect of A. auricular on AFB1-Induced Liver Injury

After a period of 7 days of adaptive feeding, 5-week-old male Sprague Dawley (SD) rats were provided with a standard diet and ad libitum access to water. The rats were then randomly assigned to 6 groups based on their body weight, with each group consisting of 6 rats. The groups included the blank control group (B), which received a normal diet without AFB1 and mycelia powder; the positive control group (P), which received a 2 mg/kg AFB1 solution via oral gavage at 9 a.m. on the first day; the high dose negative control group (HC), which received a daily oral administration of 500 mg/kg BW; the high dose mycelia powder experimental group (HT), which received an AFB1-mycelia powder solution (AFB1 2 mg/kg BW, mycelia powder 500 mg/kg BW) via oral administration at 9 a.m. on the first day; the low dose negative control group (LC), which received a daily oral administration of mycelia powder solution (100 mg/kg BW); and the low dose mycelia powder experimental group (LT), which received an AFB1-mycelia powder solution (AFB1 2 mg/kg BW, mycelia powder 100 mg/kg BW) via oral administration at 9 a.m. on the first day.

Following the aforementioned protocol, the SD rats were fed a standard diet for three additional days. After an overnight fasting period, the rats were weighed the next morning, and anesthesia was induced by injecting 0.3 mL/100 g BW of 10% chloral hydrate solution. Blood samples were promptly collected after anesthesia, with 2 mL of blood stored in 5 mL anticoagulation tubes and accelerating tubes. The samples were then transported to the hospital for liver function assessment, including ALT, AST, γ-GT, T-Bil, D-Bil, I-Bil, TP, and ALB. A portion of the liver tissue was homogenized to measure enzyme activity, including antioxidant enzymes such as superoxide dismutase (SOD), glutathione peroxidase (GST-PX), and catalase (CAT). The levels of MDA were measured following the instructions provided in the MDA kit. Additionally, liver tissue was sectioned into 10 μm slices and stained with HE (hematoxylin-eosin) for further analysis. The histopathological procedure followed the methodology described in a previous study [33]. Fresh liver tissues were carefully dissected into pieces measuring 1 cm × 1 cm. These tissue samples were then immersed in neutral formalin for a minimum of 24 h, and subsequently processed for paraffin embedding. Sections with a thickness of 10 μm were obtained from the embedded tissues. To facilitate histopathological examination, the slides were stained using hematoxylin and eosin (H&E). Microscopic analyses of the liver sections from all experimental groups were performed using a Nikon E100 microscope from Japan.

### 2.9. Statistics Analysis

The experiments were repeated three times, and date analysis was performed using SPSS 19.0 software (International Business Machines Corporation, Armonk, NY, USA); *p* ≤ 0.05 or less was considered significant.

## 3. Results

### 3.1. Characterization of AFB1 Adsorption by A. auricular Mycelia

#### 3.1.1. Assessment of Adsorption Capacity under Different Pretreatments

**Figure 1:**
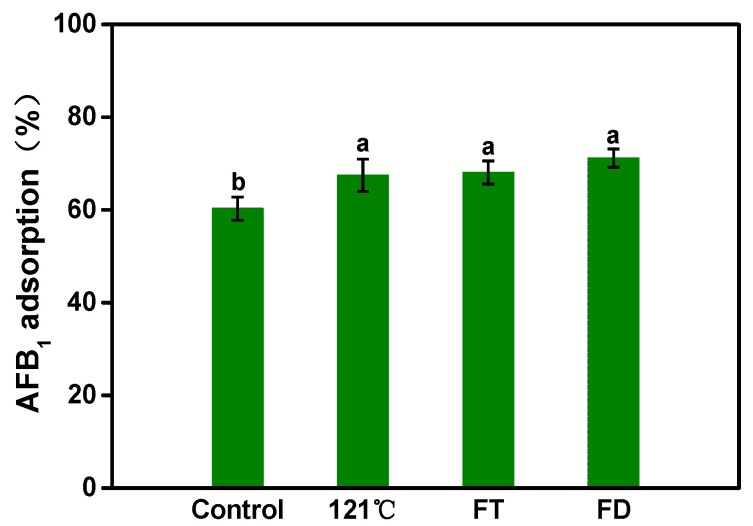
The effect of mycelia pretreatments on AFB1 adsorption. The abbreviations FT and FD correspond to repeated freeze–thaw and vacuum freeze-drying treatments, respectively, while the control group represents wet mycelia without any treatment. The values are presented as means ± standard deviation (SD); a and b indicate significant differences between different components or treatments (*p* < 0.05).

Based on the previous experimental results in our laboratory regarding the detoxification of toxins by *A. auricula*, it was observed that *A. auricular* mycelia exhibited enhanced AFB1 adsorption capability. As shown in Figure 1, compared with control group, the three different pre-treatment methods of the mycelia resulted in improved AFB1 adsorption (≤20%). However, no significant differences were observed among the three treated groups. Notably, freeze-dried mycelia demonstrated the highest adsorption rate (71.2%). Freeze-drying preserves the high biological activity of the mycelia and offers the benefits of convenient storage and processing.

#### 3.1.2. Effects of Reaction Ratio (Mycelia/AFB1), pH, Temperature and Incubation Time on AFB1 Adsorption Capacity

**Figure 2:**
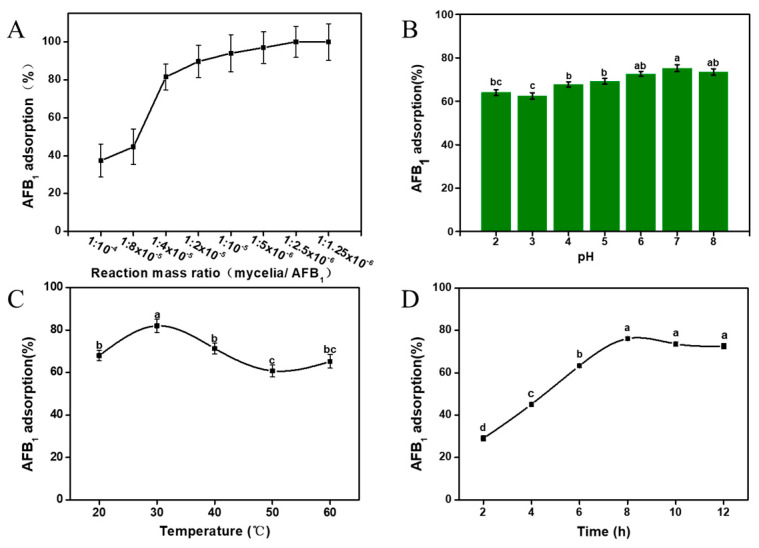
AFB1 adsorption capacity of *A. auricular* mycelia: effect of reaction mass ratio (mycelia/AFB1) (**A**), effect of incubation pH (**B**), effect of incubation temperature (**C**), and effect of incubation time (**D**). Values are expressed as means ± SD; a, b, c and d indicate significant differences between different treatments (*p* < 0.05).

Furthermore, we investigated the effects of various factors on the adsorption, including the reaction mass ratio (mycelia/AFB1), pH, temperature, and incubation time (Figure 2A–D). Figure 2A showed that, as the reaction ratio increases, the adsorption rate will show an upward trend, and will reach equilibrium under a certain ratio. When the reaction ratio (mycelia/AFB1) was 1:2.5 × 10^−6^, the adsorption quantity and adsorption rate could sustain a steady state. It showed that the adsorption has reached saturation, and the adsorption rate could still reach 81.5%.

It was observed from Figure 2B that the adsorption rate could reach the highest (75.2%) at pH 7.0, while the adsorption is less effective under acidic conditions. With the pH shifts towards alkaline, there is a slight increase in the adsorption rate, albeit within a range of approximately 20%.

The effect of temperature is shown in Figure 2C. While there were no significant variations in the adsorption rate at different temperatures, it was observed that the adsorption rate tends to be higher when the temperature approaches the human body temperature. Specifically, at 30 °C, the adsorption rate reaches 82%, surpassing the rates observed under other conditions. 

As shown in Figure 2D, the adsorption process of AFB1 by *A. auricular* mycelia exhibited a slow progression, with approximately 30% of AFB1 being adsorbed after 2 h of mixing with the mycelia. The relationship between the adsorption rate and incubation time followed a “J”-shaped growth curve. Within 8 h, the adsorption rate exhibited rapid growth, ultimately reaching equilibrium at a rate of 78.4%.

#### 3.1.3. Stability Evaluation of AFB1 Adsorption

**Figure 3:**
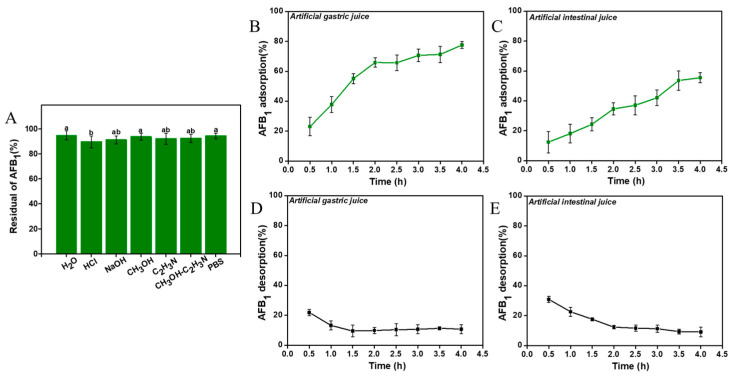
AFB1 adsorption stability of *A. auricular* mycelia: the residual AFB1 content after washing with different eluents (**A**), AFB1 adsorption and its desorption rate in artificial gastric juice (**B**,**D**), and AFB1 adsorption and its desorption rate in artificial intestinal juice (**C**,**E**). Values are expressed as means ± SD; a, b, and ab indicate significant differences between different treatments (*p* < 0.05).

The stability evaluation of the adsorption was shown in Figure 3. There was no significant difference in the desorption capacity between different eluents (Figure 3A). Following elution with seven different eluents, the residual AFB1 content in the compound was between 89.6% and 94.8%, and the elution of AFB1 to the binders were all less than 11%. The results indicated that the binders were stable.

In simulated vitro experiments, we found that with the extension of the treatment time, the AFB1 adsorption rate gradually increased, indicating that the mycelia powder has the capability to adsorb AFB1 in gastric juice and intestinal juice (Figure 3B,C). This suggests that *A. auricular* mycelia can reduce the bioavailability of AFB1 through adsorption. Our findings demonstrate the stability of the mycelia powder–AFB1 binders in the in vitro environment. From Figure 3D,E, the addition of artificial gastrointestinal juice resulted in minimal desorption of AFB1 from the binders. Hence, we believe that *A. auricular* mycelia has promising potential for application in the prevention and control of AFB1 *in vivo*.

### 3.2. Analysis of Adsorption Behavior

#### 3.2.1. Adsorption Kinetics

**Figure 4:**
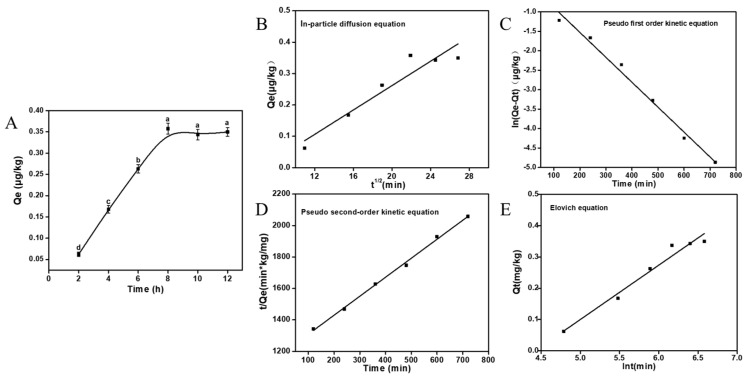
Kinetics of adsorption: the kinetics curve of AFB1 adsorbed by *A. auricula* mycelia (**A**–**E**) represent the kinetics model fitting curve of AFB1 adsorbed by *A. auricula* mycelia, using four kinetic models including the intra-particle diffusion model, pseudo-first-order kinetic model, pseudo-second-order kinetic model, and Elovich model. Values are expressed as means ± SD; a, b, c, and d indicate significant differences between different treatments (*p* < 0.05).

The curve of the adsorption amount of *A. auricular* mycelia for AFB1 with adsorption time is showed in Figure 4A. The adsorption amount exhibited an increasing trend from 2 to 8 h, reaching equilibrium at 8 h (0.358 μg/kg). To analyze the adsorption process, four kinetic models, namely the intra-particle diffusion model, pseudo-first-order kinetic model, pseudo-second-order kinetic model, and Elovich model, were employed.

Figure 4B–E shows the fitting line of the kinetic models, and the four model fitting parameters are listed in Table 1. The pseudo-second-order kinetic equation exhibits a significantly higher fitting value (0.9976) compared to the other models, suggesting that it provides a better description of the adsorption process of AFB1 by *A. auricular* mycelia.

#### 3.2.2. Adsorption Isotherm

**Figure 5:**
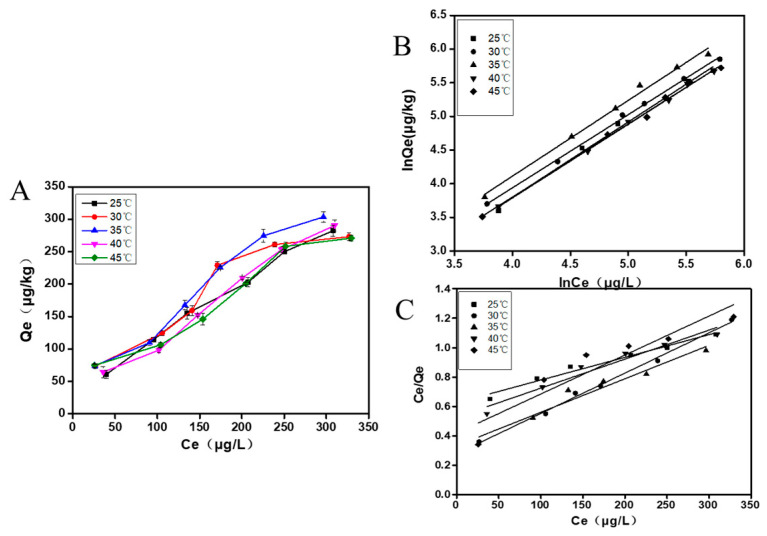
Adsorption behavior of *A. auricula* mycelia on AFB1: the adsorption isotherm of AFB1 (**A**), the adsorption isotherm of Freundlich equation fitting AFB1 adsorbed by *A. auricula* mycelia (**B**), and the adsorption isotherm of Langmuir equation fitting AFB1 adsorbed by *A. auricula* mycelia (**C**). Values are expressed as means ± SD.

The adsorption isotherm of Qe (adsorption amount of mycelia) with Ce (equilibrium concentration of AFB1) is shown in Figure 5A. The equilibrium adsorption amount of mycelia demonstrates a significant increase with the rise in the initial concentration of AFB1. At the same time, the adsorption of AFB1 by mycelia showed a decreasing trend as the temperature increased from 25 °C to 45 °C, with the highest adsorption observed at 35 °C. Furthermore, we used the Freundlich equation and the Langmuir equation to fit data of adsorption isotherm.

The fitting line of the isothermal adsorption models were shown in Figure 5B,C, and the corresponding parameters are listed in Table 2 and Table 3. The analysis revealed that the Freundlich model was more suitable for describing the adsorption isotherm, due to its high R^2^ (R^2^ > 0.99 at different temperatures). The results suggest that the adsorption process of AFB1 by *A. auricular* mycelia follows the heterogeneous adsorption of multi-molecular layers, as described by the Freundlich equation.

#### 3.2.3. Adsorption Thermodynamics

Adsorption thermodynamic parameters are listed in Table 4. It can be observed that, as the temperature increases, the value of Koc first rose and then decreased. It indicated that the adsorption of *A. auricular* mycelia on AFB1 increases with temperature, exhibiting a trend of first rising and then falling. These findings are consistent with the results obtained from the previous fitting of the Freundlich equation.

According to ΔG < 0, it can be judged that the adsorption process is spontaneous and is affected by temperature. The absolute value of ΔG initially increases and then decreases as the temperature rises. This indicates that the adsorption process reaches its maximum at 40 °C, and slows down beyond that temperature.

It can be judged by ΔH = −6.31 that the adsorption reaction process is exothermic. It suggests that an increase in temperature is not favorable for the progress of the reaction. This is consistent with the previous single-factor result of incubation temperature, and in line with the result of ΔG < 0.

The ΔS value represents the variation in adsorption entropy, reflecting the degree of disorder between the solid and liquid phases during the reaction. ΔS > 0 values indicate an increase in disorder. With increasing temperature, the ΔS value initially rises and then decreases. This trend suggests that higher temperatures have a beneficial impact on the reaction progress, promoting increased disorder. However, once the maximum value is reached, the adsorption reaction proceeds unfavorably, leading to a decrease in disorder.

### 3.3. Evaluation of the Intervention Effect of A. auricular on AFB1-Induced Liver Injury

In this study, *A. auricular* mycelia showed an excellent ability to adsorb AFB1 *in vitro*. To investigate the effect of AFB1 adsorption by mycelia *in vivo*, SD rats were fed with mycelia, AFB1, or a mixture of both. The results showed that the addition of mycelia improved liver function and restored antioxidant levels in SD rats. Overall, *A. auricular* mycelia exhibited a protective effect against AFB1-induced liver damage.

**Figure 6:**
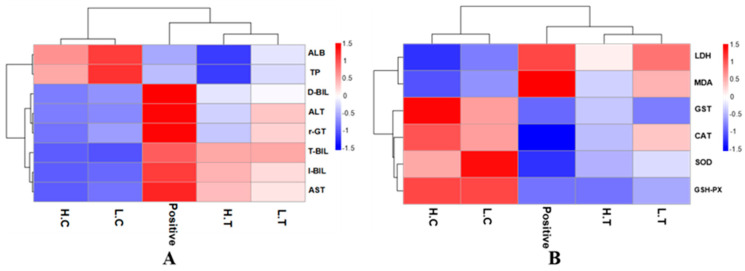
Heat map of liver functions, lipid peroxidation and antioxidant levels. Positive (P) represents the positive control group, rats infected with AFB1; LC and HC represent low-dose *Auricularia auricula* mycelia-fed and high-dose mycelia-fed normal SD rats, respectively; LT and HT represent the rats treated with mixed toxins and different doses of mycelia powder. (**A**) liver function related index; (**B**) lipid peroxidation and antioxidant levels.

The assessment of liver function was conducted, and the findings are presented in Figure 6A. There were significant differences between the negative control group, the positive control group, and the experimental group, as can be seen in Figure 6A. Obviously, rats in the positive control group exhibited abnormal liver function indicators due to AFB1 induction. The levels of TP and ALB significantly decreased, indicating a decline in liver reserve function. The elevated levels of ALT and AST suggested severe liver cell damage. The varying levels of T-Bil, D-Bil, and I-Bil indicated impairment in liver excretory function. The presence of high γ-GT levels in the positive control group, which is typically produced by the liver, indicated abnormal or pathological liver conditions. In summary, AFB1 infection led to abnormal liver function in the experimental rats. However, the experimental group showed mitigated liver damage compared to the negative control group. Notably, this protective effect was observed to be dose-dependent on the ingestion of mycelia powder.

Similarly, the protective effect of mycelia on SD rats is also evident in Figure 6B. In the positive control group, an increase in MDA and LDH levels was observed, indicating AFB1-induced lipid peroxidation in the liver of rats. The levels of GST, CAT, SOD, and GSH-PX, which are associated with *in vivo* oxidative status, significantly decreased. This reduction indicated a decrease in the antioxidant capacity of rats, and an imbalance in the regulation mechanism of endogenous enzymes or non-enzymatic factors. Such conditions are unfavorable for rats to counteract external substance interference. Conversely, in the experimental group of rats fed with a mixture of mycelia, a significant increase in the activity of endogenous antioxidant enzymes or non-enzymatic factors was observed, accompanied by a decrease in MDA and LDH levels. These findings suggest that *A. auricular mycelia* alleviated AFB1-induced lipid peroxidation in SD rats and prevented the accumulation of reactive oxygen species.

**Figure 7:**
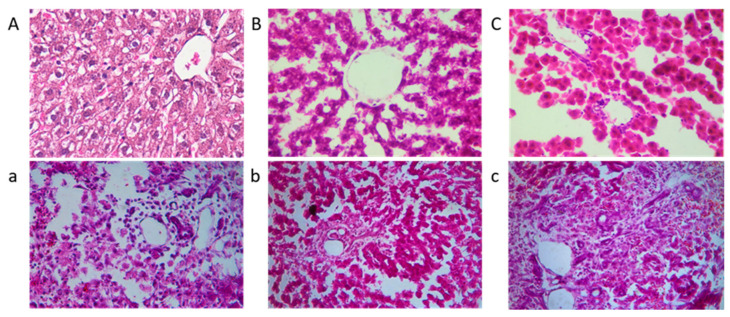
The liver histopathological slices observation of rats; (**a**–**c**) represent the positive group, the low dose experimental group, and the high dose experimental group, respectively, and (**A**–**C**) are their corresponding control groups.

Figure 7 presents histological sections of rat livers stained with HE. The liver structure in the control group (A, B, and C, without AFB1) appeared normal, with no significant differences observed among A, B, and C. These findings indicate that the addition of mycelia powder does not induce liver damage, and is considered safe. In contrast, the positive control group exhibited evident lesions, characterized by pronounced congestion of hepatic sinusoids and a light purple appearance throughout the section. Hepatocytes showed disordered arrangement, nucleus loss, cytoplasmic outflow, and noticeable infiltration of inflammatory cells in the portal area. In liver tissue sections b and c, the overall liver structure was mostly normal, with some remaining nucleus loss. However, the pathological changes were significantly alleviated, with section c demonstrating a liver state closer to normal. In summary, *A. auricular* mycelia effectively mitigated liver damage caused by AFB1, and the protective effect improved with increasing mycelia dosage. Moreover, when fed mycelia powder alone, it did not induce liver damage and exhibited a certain level of liver protection.

## 4. Discussion

Due the high toxicity and exposure risk associated with AFB1, many methods are developed to mitigate its adverse effects. Among them, the use of microbials to remove AFB1 from materials has garnered significant attention, particularly in the feed industry [34]. A lot of microbial species, such as *Lactobacillus rhamnosus*, *Accharomyces cerevisiae*, and *Leurotus eryngii*, have been identified for their ability to remove AFB1, achieving an average clearance rate of 86% [19,35,36,37]. Interestingly, we discovered that the mycelia of *A. auricular*, a popular edible fungus widely cultivated in Southeast Asia and recognized for its potential pharmaceutical value, exhibited a higher efficiency in removing AFB1, with a clearance rate of 88.2% [38,39,40]. Under optimal conditions, the AFB1 clearance rate reached 98.3%. Therefore, *A. auricular* mycelia offer a promising, safe, and appealing approach to combat aflatoxin contamination in food [41,42]. This study was designed to investigate the adsorption effect of *A. auricular* mycelia on AFB1, establish its safety, and explore the underlying mechanism.

This study provides evidence that *A. auricular* mycelia exhibit robust adsorption capacity for AFB1. The adsorption rate remains consistently high, exceeding 60%, at both lower (20 °C) and higher (121 °C) temperatures. The influence of pH on adsorption is minimal, with a minimum adsorption rate of 60%. Alkaline conditions favor adsorption, with a notable adsorption rate of 75.2% at pH 8. Furthermore, despite a decrease in the adsorption rate in artificial intestinal juice and artificial gastric juice, it still reaches 55.5% and 77.6%, respectively, confirming the ability of *A. auricular* mycelia to adsorb AFB1 within the human gastrointestinal tract. In the case of AFB1-poisoned rats, *A. auricular* mycelia significantly alleviated liver damage and reduced oxidative stress and lipid peroxidation levels. Moreover, the adsorption process demonstrates remarkable stability, with the highest desorption rate observed at 10.4% after treatment with organic reagents. In artificial intestinal juice and gastric juice, the binder maintains high stability, exhibiting desorption rates of 10.7% and 9.1%, respectively.

Based on the findings from our experiment, we proposed the mechanism of *A. auricular* mycelia against AFB1-induced liver damage. In addition to its direct role in reducing AFB1 adsorption in the gastrointestinal tract, similar to other enterosorption therapies, *A. auricular* mycelia also exhibit the ability to attenuate AFB1-induced oxidative stress upon entering the body, and to enhance the activity of phase II detoxification enzymes. The freeze-dried mycelia demonstrate a higher adsorption rate, which can be attributed to their elevated mycelial activity, and they are also more convenient for storage and application in food processing [43]. Owing to the high stability of this adsorption, different interpretations of adsorption behaviors emerged. According to the adsorption kinetic curve and thermodynamic research, we consider this adsorption as a form of steady chemical adsorption. However, the adsorption process aligns with empirical formulas, indicating a characteristic of heterogeneous multi-molecular layer adsorption, typically associated with physical adsorption. Considering the porosity and complexity of *A. auricular* mycelia, we propose that the adsorption process involves two phases: physical adsorption and chemical adsorption.

The hepatoprotective effects of *A. auricular* mycelia on rats have been successfully validated, confirming our initial hypothesis that *A. auricular* mycelia can alleviate AFB1-induced oxidative stress reactions and promote liver recovery. What sets it apart is that, in addition to post-digestion detoxification, *A. auricular* mycelia can directly reduce the adsorption of AFB1 in the gastrointestinal tract, thereby intercepting the initial step of toxin metabolism. Interestingly, the addition of *A. auricular* mycelia not only exhibited no toxicity in normal rats, but also provided favorable conditions for their growth, while exerting a protective effect on the liver. We speculate that this could be attributed to the nutritional and medicinal value inherent in *A. auricular* mycelia.

## 5. Conclusions

*A. auricular* mycelia exhibit the ability to adsorb AFB1 and mitigate AFB1-induced liver damage in rats. Its liver-protective effect is attributed to multiple mechanisms, including a reduction in AFB1 bioavailability through stable adsorption, attenuation of AFB1-induced oxidative stress, and enhancement of phase II detoxification enzyme activity. Furthermore, *A. auricular* mycelia contribute to the normalization of liver function indicators. The adsorption process is considered to involve both physical and chemical adsorption phenomena. In conclusion, *A. auricular* mycelia demonstrate the capability to adsorb AFB1, thereby alleviating AFB1-induced liver damage and providing liver protection. This adsorption ability remains effective even under extreme conditions, suggesting its potential for industrial applications. With its edibility and medicinal value taken into account, *A. auricular* mycelia hold promise as a potential food supplement or health-care product for the ultimate prevention of AFB1 and the promotion of liver health.

The research conducted in this study provides theoretical evidence for the edible fungi removal of AFB1. However, the adsorption of AFB1 by *A. auricula* is a complex biological reaction, and further investigation is needed to elucidate the adsorption mechanisms. Specifically, this study examined the adsorption of AFB1 by different *A. auricula* tissue structures. To further understand the adsorption mechanisms, a more in-depth investigation of the binding sites for AFB1 adsorption by *A. auricula* is warranted. Additionally, in order to clarify the impact of *A. auricula* on AFB1 adsorption and metabolism, future research should incorporate *in vivo* and *in vitro* experiments to investigate the adsorption of AFB1 in the intestine, the formation of biomarkers and parent compounds within the organism.

## Figures and Tables

**Table 1:** The kinetics model fitting parameters of AFB1 adsorbed by *A. auricular*.

Fitting Model	Formula	R^2^
In-particle diffusion equation	Qt = 0.06 t^0.5^	0.8870
Pseudo first-order kinetic equation	ln(q_e_ − q_t_) = lnq_e_ − 0.16 t	0.9856
Pseudo second-order kinetic equation	t/qt = 1/13.77 qe^2^ + 1/qe	0.9976
Elovich equation	Qe = 0.09 + 0.02 lnt	0.9618

**Table 2:** The adsorption isotherm parameters of the Freundlich equation fitting AFB1 adsorbed by *A. auricular*.

Temperature (K)	K_f_	1/*n*	Freundlich Formula	R^2^
298	0.11	0.02	Qe = 0.11 × Ce0.02	0.9981
303	0.22	0.04	Qe = 0.22 × Ce0.04	0.9927
308	0.24	0.05	Qe = 0.24 × Ce0.05	0.9901
313	0.17	0.03	Qe = 0.17 × Ce0.03	0.9951
318	0.11	0.02	Qe = 0.11 × Ce0.02	0.9979

**Table 3:** The adsorption isotherm parameters of the Langmuir equation model.

Temperature (K)	K_L_	Langmuir Formula	R^2^
298	0.01	Qe = 0.278 × Ce/(1 + 0.01 Ce)	0.9943
303	0.02	Qe = 0.625 × Ce/(1 + 0.02 Ce)	0.9680
308	0.04	Qe = 0.333 × Ce/(1 + 0.04 Ce)	0.9462
313	0.09	Qe = 0.419 × Ce/(1 + 0.09 Ce)	0.8551
318	0.04	Qe = 0.527 × Ce/(1 + 0.04 Ce)	0.9464

**Table 4:** The thermodynamics parameters of AFB1 adsorbed by *A. auricular*.

Temperature (K)	Koc	∆G (KJ/mol)	∆H (KJ/mol)	∆S (KJ/mol)
298	10.59	−22.43	−6.31	0.05
303	21.59	−21.35	−6.31	0.07
308	24.38	−36.85	−6.31	0.10
313	17.27	−50.39	−6.31	0.11
318	11.13	−43.82	−6.31	0.09

## Data Availability

The data used to support the findings of this study can be made available by the corresponding author upon request.
